# HEARING AND VISION LOSS: EPIDEMIOLOGIC AND INTERVENTION IMPLICATIONS FOR SOCIAL ISOLATION, LONELINESS, AND MOOD DISORDERS

**DOI:** 10.1093/geroni/igad104.0527

**Published:** 2023-12-21

**Authors:** Alison Huang, Roger O’Sullivan, James Lubben

## Abstract

This session will discuss hearing loss and vision impairment as modifiable risk factors for social isolation, loneliness, and mood disorders from both epidemiologic and intervention perspectives. Prevalence of hearing and vision loss is high; two-thirds of older adults have hearing loss and over one-quarter have vision impairment. Hearing loss and vision impairment have been linked to poor cognitive and physical health but remain understudied in the context of social and mental health in large, population-based studies. Research gaps remain in measurement (findings typically based on subjective assessments) and generalizability. This session will investigate associations between objectively measured hearing, vision, and dual sensory loss on social isolation and mood disorders using a sample nationally representative of older adults in the US. This session will also describe potential mechanistic and environmental factors, such as fatigue and exclusion of hearing loss and vision impairment in health care settings that may explain observed associations. Whether treatment of hearing loss and vision impairment is an effective intervention for improving social and mental health is unknown. We will conclude by presenting findings from a randomized controlled trial designed to test whether a hearing intervention consisting of provision of hearing aids and related technologies, counseling, and education could reduce cognitive decline in older adults with untreated hearing loss. We focus on the effect of hearing intervention on social isolation and loneliness, both pre-specified outcomes of this trial. This is a Loneliness and Social Isolation Interest Group Sponsored Symposium. This is a collaborative symposium between the Loneliness and Social Isolation and Sensory Health Interest Groups.

